# Attitudes towards Green Urban Space: A Case Study of Two Italian Regions

**DOI:** 10.3390/ijerph18126442

**Published:** 2021-06-14

**Authors:** Alessia Grigoletto, Mario Mauro, Francesco Campa, Alberto Loi, Maria Cristina Zambon, Marzia Bettocchi, Mark Nieuwenhuijsen, Laura Bragonzoni, Pasqualino Maietta Latessa, Stefania Toselli

**Affiliations:** 1 Department of Biomedical and Neuromotor Sciences, University of Bologna, 40126 Bologna, Italy; alessia.grigoletto2@unibo.it (A.G.); stefania.toselli@unibo.it (S.T.); 2 Department of Basic Medical Sciences, Neuroscience and Sense Organs, University of Study of Bari, 70121 Bari, Italy; 3 Department of Life Quality Studies, University of Bologna, 47921 Rimini, Italy; francesco.campa3@unibo.it (F.C.); laura.bragonzoni4@unibo.it (L.B.); pasqualino.maietta@unibo.it (P.M.L.); 4 Department of Pharmacy, Biotechnology and Sport Science, University of Bologna, 40126 Bologna, Italy; alberto.loi10@studio.unibo.it; 5 Welfare Area and Promotion of Community Wellness, Bologna Municipality, 40126 Bologna, Italy; cristina.zambon@comune.bologna.it (M.C.Z.); marzia.bettocchi@comune.bologna.it (M.B.); 6 ISGlobal, 08036 Barcelona, Spain; mark.nieuwenhuijsen@isglobal.org

**Keywords:** attitude, green space, health promotion, park

## Abstract

Green spaces are defined as open spaces of ground, covered by vegetation, including parks and gardens. This kind of environment is linked to many positive effects and its importance is growing due to increasing urbanization. Understanding what drives people to use green urban space is fundamental to creating appropriate campaigns to develop the use of such spaces and improve the citizens’ quality of life. A questionnaire on the attitude towards green space was developed and submitted to people from two Italian regions. Emilia-Romagna and Veneto are two regions in the North of Italy with different territorial policies. Three hundred and ten surveys were collected (167 in Emilia-Romagna and 143 in Veneto). Significant differences were observed between regions, age groups and in relation to the kind of work (*p* < 0.05). People from Emilia-Romagna have higher scores of attitudes towards green space than people from Veneto, underlining the importance of territorial policies. Moreover, younger participants (18–30 years) seem to be less attracted to green urban space. Being an employee seems to influence the attitude towards green space. Particular attention should be given to subjects of the younger age groups and to the number of hours spent at work. This could be an important element for future research, so that political action can be implemented with these categories in mind.

## 1. Introduction

Urbanization has led to significant changes in the landscape, with more people concentrated in cities [1]. It constitutes a massive environmental transformation in which natural ecosystems are largely lost or degraded, resulting in a reduction of the possible beneficial effects that nature can provide to people, including those related to health and psychophysical well-being. This undoubtedly has a strong impact on public health, since it is expected that by 2030 three out of every five people of the worldwide population will live in an urban area [1]. Therefore, one of the most important challenges for the future will be to create people-friendly cities, and the safeguard of green spaces represents a fundamental aspect to achieving this. Cities are usually relatively nature-poor due to the great range of competing land-use [2] or, are areas in which urban natural spaces face considerable development pressure [3]. Green spaces are defined as the open spaces of ground, partially or completely covered by vegetation, including parks and gardens. The loss of these kinds of spaces likely leads to less contact with the natural world for many urban dwellers [4,5], a circumstance that has potential negative impacts on the quality of life and well-being of the population [6]. Furthermore, certain environmental factors which characterize urban settings, such as air pollution, noise and extremely high temperatures have been associated with increased mortality [7,8,9], and the protection/creation of natural outdoor environments might help reduce these negative environmental factors and their impact on health and life-expectancy [10,11]. In addition, the exposure to and the interaction with nature have been shown to have a role in cognitive function and social cohesion, and to have long-lasting psychological benefits [12,13,14,15,16]. Furthermore, urban green spaces provide areas for recreation, community activities and physical activities; the latter, in particular, has a significant protective role in cardiovascular disease, diabetes and obesity [17,18,19]. Time spent in a natural setting has been linked to reduced stress [20] and to improved concentration [4,21]. In addition, the access to green space and parks and the proposal of physical activity programs may also be a protective factor for the disadvantaged populations. Various ethno-racial groups exhibit distinct preferences for leisure settings and have diverse reasons for visiting recreational facilities such as parks. Dahmann et al. reported that the recreational programs offered, and their variety, may be restricted in lower-income communities with fewer fiscal resources; thus, urban recreation programs that involve varying degrees of physical exertion should be implemented in these communities [4]. In fact, poor people and non-white persons have a lower possibility to access the park with a consequent higher health risk associated with a lack of physical activity [22].

Different types of mechanisms have been proposed to explain the beneficial effects of a green environment: (1) restoration theory, according to which watching a green space influences health perception and well-being, because of the intrinsic quality of the natural outdoor environment [7,10,21]; (2) biodiversity increase, which concerns the link between green space in terms of a healthy environment, influences the immune response, and is characterized by lower temperature and lower air and noise pollution [7,8,9,22,23,24,25,26,27]; (3) opportunity to perform physical activity, by promoting leisure walking, walking through the space when running errands, active playing and sports [10,11,27,28,29,30]; (4) enhancement of social interaction and improved social cohesion in the community [30,31,32,33]. However, it should be added that although the beneficial effects of natural habitats are commonly reported, the negative effects should also be considered [34]. According to Dudek et al. there could be a worsening of the health risk among allergy sufferers who visit forest areas or their immediate vicinity [35]. The authors suggest that information on the risk of allergenic pollen in natural communities would be extremely useful for visitors, since visiting appropriate places in the forest allows the exposure of allergen sensitive people to be reduced. 

Even though there has been a growth in the literature focused on the importance of green urban spaces and several studies have highlighted a relationship between exposure to the natural environment and better health perception [36], the mechanism that brings people to use green space is still unclear [37,38]. In particular, some concerns involve the optimal distance of the house from the park to ensure a frequent use of green spaces. The current recommended distance between a residence and the nearest open public space is 300 m [32]; however, other studies have suggested that people are willing to walk for even longer distances in order to have access to a green urban space if parks have some attractive features [39,40]. Other factors that may influence access to parks are socio-economic status, sex and age. Previous studies have shown that the use of natural environments may differ according to socio-economic status and sex [36,41,42,43]: women between 18 and 30 and those over 65 generally showed a higher frequency of use in comparison with men or people belonging to other age groups [44]. In addition, women are disproportionately affected by common mental health issues and they are more sensitive to the neighbourhood environment [45]. Women are therefore a group for which the effects of urban parks could be especially important [46]. With regard to age, young people generally underutilize parks and green urban spaces because they prefer to spend their leisure time in other kinds of activities rather than in outdoor experiences [47].

Another aspect concerns the link between physical activity and park use. Physical inactivity is one of the five leading global risks for mortality in the world, because it is responsible for raising the risk of chronic disease and cancers [48]. It is estimated that 3.3 million people die annually worldwide as a result of physical inactivity and a significant proportion of the adult population remains inactive [21,27]. The exposure to a natural environment is linked to triggering a higher amount of physical activity among residents, and a lower mortality rate [31,32,40]. Some studies suggest that peoples’ perception of the environment could influence the willingness and intention to use the surrounding environment [49,50]. Relatively few studies have assessed the effects of the perception of green space and the use of parks in terms of health promotion and such studies are lacking in Italy. Encouraging park visitation could promote the physical and mental health of residents. Even if there is increasing literature about the importance of green spaces, the results are contrasting because of the different measurements, statistical analysis and covariate sets used. Thus, the relationships change based on the individual characteristics considered, and the question of what drives people to use green urban spaces is still open.

Therefore, the first aim of this exploratory research was to develop a questionnaire to evaluate the attitude towards green spaces and understand what drives citizens to use them. In Italy, different policies regarding green spaces are adopted among regions [51,52].

Thus, a second aim was to assess whether there are any differences in attitudes toward parks between the inhabitants of two Italian regions: Emilia-Romagna and Veneto. These are two bordering regions in the north of Italy, similar in population characteristics and socio-economic status. Emilia-Romagna has an area of 22,444 km^2^ with a population of 4,459,477 people, mostly living in the provincial capitals, and a pro capita income of 22,500 euros. The area of Emilia-Romagna is mainly covered by plains and mountains with rainfall ranging from 600 mm per year in the plain to 1500 mm per year in the mountainous area. There are two national parks in this region covering an area of 36,000 hectares. Veneto is further north than Emilia-Romagna and has an area of 18,390 km^2^ mainly covered by plains and mountains. There are 4,905,854 people who live in Veneto and most of them live in the provincial capitals, with a pro capita income of 21,994 euros. The rainfall range is very similar to Emilia-Romagna. Veneto has only one national park, but it covers an area of 32,000 hectares [53,54,55,56].

However, as regards physical activity programs in green spaces for the population, the two regions show marked differences. In Emilia-Romagna, a project to increase green areas in cities by 20% was approved in 2020, while in Veneto there are no similar projects.

In March 2021, the Emilia-Romagna region allocated 4.5 million euros for parks and biodiversity agencies to promote sustainable development and to combat climate change, and 3.6 million euros for projects that protect and enhance the natural environment and the environmental heritage throughout the whole region [57,58]. At the same time, eight areas of Emilia-Romagna were chosen by the Europarc Federation to participate in the “European charter for sustainable tourism” with the goal of developing environmental tourism [59]. Since 2005, the municipality of Bologna (the regional capital) has celebrated “Arbor Day”, where trees are planted in different parts of the city [60]. In Bologna, there have been other initiatives to improve the use of green urban spaces. In fact, since 2010 Bologna citizens have had the possibility to experience different kinds of outdoor physical activity in parks in the summertime through the project “Parchi in movimento” [61]. Moreover, from 2011 onwards, senior citizens in Bologna have had the opportunity to be physically active while also improving their socialization thanks to the city’s project “Badabene alla salute”, which envisages twice weekly physical activity group sessions and walking groups in public parks and other urban outdoor spaces located in the six districts of Bologna. Similar projects were carried out in the other provinces of Emilia-Romagna. Veneto has had only a few projects regarding green spaces and these are linked to local municipalities (such as Padua or Verona); there were no similar regional projects or events [62,63]. Higher scores in attitude towards green urban spaces could be a demonstration of the efficacy of the territorial policies in Emilia-Romagna.

Therefore, the first objective of the present study was to propose a questionnaire that could be effective for understanding attitudes towards green space. Many factors (demographic, proximity and park use) may affect attitudes, and in literature these were often considered individually. Therefore, the second goal was to gain a better understanding of the influence of these factors, and assess the differences in the attitude towards green spaces, not only at a territorial level, but also considering sexes, age groups, level of education, distances between home and green space and among people who use parks for physical activity or not. In particular, in Italy there is a lack of studies about attitudes towards green urban space, and this preliminary study has the potential to provide helpful, if not generalizable, information on this matter. The understanding of what components influence the determination of an attitude could be very useful for managers and decision makers engaged in public health, as this could help guide management strategies.

## 2. Materials and Methods

### 2.1. Participants

Three hundred and fifty subjects completed the questionnaires, but some were excluded from the analysis as they lacked important information. Ultimately, 310 surveys contained all the information and were considered in the present study: 167 from Emilia-Romagna and 143 from Veneto. The survey was approved by the Bioethics Committee (prot. N. 022254) and was administered in the two regions both on paper (distributed in parks) and in an online version with Google Moduli. Distribution of the survey began in May 2020 and the online version was closed in September 2020. 

In addition, before administering the questionnaire a pre-test was carried out using a small sample of respondents to assess its reliability. For this purpose, we recruited 60 subjects via a convenience-based sampling to validate the survey. This group consisted of 30 participants from Emilia-Romagna and 30 from Veneto, divided equally between men and women, and age groups. The questionnaire was administered in the paper version to people who were randomly recruited in both regions using a social network, such as Facebook. 

### 2.2. Procedures

A new questionnaire was developed to investigate the attitude that drives people to use urban parks. Attitude represents a synthetic assessment of a psychological object evaluated in positive or negative dimensions [46,47]. The survey was divided into two subsections: (1) demographic information, and (2) attitude toward green space components. The first section collected demographic information, including sex, date and place of birth, region and city of living, level of education, marital status, occupation and the distance from their home to the nearest urban park. Information regarding the use of parks for physical activity was also gathered. The second part included questions designed to assess participants’ attitude toward green urban spaces [64]. The statements were evaluated using the Likert scale. 

The questionnaire included fifteen items, divided into three components: cognitive, behavioural and affective. Questions one to five belonged to the cognitive component, which can be measured through the belief types of value orientations, objective knowledge and perceived outcomes. The second component covered the behavioural aspect, and investigated park use and the participation in outdoor nature recreation. The items included in this section were from number six to number ten. Finally, questions from eleven to fifteen examined the affective component, based upon basic emotions differentials. All three components were evaluated with the Likert scale, from 1 to 5, in which 1 meant “strongly disagree” and 5 “strongly agree”. The data used for this statistical study will be available from the corresponding author upon request. 

### 2.3. Statistical Analysis 

In order to assess the questionnaire’s validity, its internal consistency was evaluated by Cronbach’s alpha coefficient on the answers of the recruited pre-test sample; a confirmatory analysis (CFA) for the convergent validity of the constructs was then performed. Cronbach’s alpha was considered reliable for values between 0.5 and 0.9.

Subsequently, to better achieve the objectives of the study, the suitability of the sample size was assessed using the G-Power software 3.1.9.2. An a priori power analysis was conducted to ensure that the number of participants was representative for the purposes of this study. To identify the sample size for the study, we assessed an a priori: computer required sample size given α, power and Effect Size by G*Power (version 3.1.9.2, Universitat Kiel, Kiel, Germany). When ANOVA was selected (α = 0.05; 1 − β = 0.90; effect size f = 0.25) a sample size of 270 participants was detected. When multiple regression was selected, the calculated outcomes parameters detected a sample size of 130 participants. Additional subjects were involved to ensure the availability of data in case of problems with data collection.

Variables’ normality was verified with the Shapiro–Wilk test. Descriptive statistics (means and SD) and frequencies were calculated. Since the variables were not normally distributed, a non-parametric ANOVA was used to assess differences between regions, sex, age groups, marital status, education level, distance from the park and use of the park. When a significant F ratio was obtained, the Tukey post hoc test was used to evaluate the differences among the groups. As regards demographic factors of proximity and park use, the differences of the frequencies between the two regions were assessed by the Chi-square test.

Finally, to further understand the influence of demographic characteristics on green space perception, a set of multiple regression models was built. A backward multiple regression analysis was carried out to assess possible predictors of the total score obtained in the three different components. Some demographic and personal characteristics were used as independent variables. In particular, age groups, sex, marital status, region of living, educational level, profession, distance from the park and use of the park were included in the model. Predictors inputted into the model were those found to have significant associations with the total score obtained in the three different components (i.e., *p* < 0.05), while those with *p* > 0.05 were removed from the model. After performing the model, all the hypotheses were verified.

A data analysis was performed using Statistica for Windows, version 8.0 (Stat Soft Italia srl, Vigonza, Padua, Italy).

## 3. Results

### 3.1. Validation of the Questionnaire

The Cronbach’s α value was 0.888, and the Cronbach’s alpha values of the different components of attitude were all above the threshold of 0.7, which can be regarded as reliable. Loading values, used to assess the relationship between variables, ranged from 0.583 to 0.965 among the different items in this study. Since the alpha was 0.929 for the cognitive component, 0.704 for the behavioral component and 0.761 for the affective component, the questionnaire could be considered valid. The model derived from the confirmatory factor analysis showed a fit with the data (Minimum discrepancy per degree of freedom, CMIN = 70.08; df = 51, CMIN/df = 1.06; Comparative Fit Index, CFI = 0.961; Root mean square residual, RMR = 0.03). According to conventional criteria, the Chi-squared/df < 2, CFI > 0.9 and RMR < 0.05 indicated a good fit [65,66,67].

Table 1 shows the results of Cronbach’s α for the sample of sixty people.

### 3.2. Assessment of the Attitude toward Green Space

#### 3.2.1. Demographic and Socio-Economic Characteristics

Table 2 summarizes the demographic and socio-economic characteristics of the subjects that participated in the study: 167 subjects (54%) come from Emilia-Romagna and 143 (46%) from Veneto. Most of the respondents were female (*n* = 194, 62%). To consider the representation of subjects according to age, people were divided into 10-year age class groups: the class most represented was the 51–60 years group (*n* = 81, 27%), followed by the 18–30 years (*n* = 66, 21%), 41–50 (*n* = 51, 16%), 31–40 (*n* = 49, 16%), 61–70 (*n* = 46, 15%) and the over 70 (*n* =14, 5%). Most of the participants had gained a high school diploma (*n* = 106, 35%) or a master’s degree (*n* = 108, 35%). A large part of the sample lived at a distance of less than 300 m from a park (*n* = 213, 69%). Park users numbered 206 (67%) and non-users 104 (33%). 

Significant differences between the participants of the two regions were observed for certain demographic characteristics, such as age groups, education levels, marital status and distance from the park (*p* < 0.05). In Emilia-Romagna, most participants were in the age group 18–31 years for men (22.4%) and 51–60 years (27.5%) for women. In Veneto, the situation was similar for women (28.51% in the age group 51–60) but different for men, where the highest percentage of participants was observed in the age groups of 31–40 years and 51–60 years (23.7%). Significant differences were also observed in the education level: a generally higher level was observed in Emilia-Romagna compared to Veneto. Married people were more represented than those with the other statuses (50.0% for men and 51.4% for women in Emilia-Romagna, and 61.0% for men and 42.9% for women in Veneto). Even though in both regions the people who lived at a distance of less than 300 m from the park were higher than those who lived farther (82.8% for men and 70.6% for women in Emilia-Romagna, and 67.8% for men and 57.1% for women in Veneto), the difference between the two regions was significant. No significant differences were observed between regions in users for physical activity and non-users: users were more numerous than non-users, with the highest percentage of men in Emilia-Romagna (74.1%).

Participants were asked if they regularly used the park, since this information could influence their attitude toward green spaces: 67% of the participants regularly used the parks (*n* = 206), while 33% (*n* = 104) did not. Of this percentage, 47% (*n* = 96) of users lived in Veneto and 53% (*n* = 110) in Emilia-Romagna; 60% (*n* = 123) of users were female and 40% (*n* = 83) were male. In Veneto, the percentage of women that used the park was 65% (*n* = 55) and the percentage of men users was 66% (*n* = 39). Regarding non-users, women represented 35% (*n* = 29) and men 34% (*n* = 20). In Emilia-Romagna, women users were 62% (*n* = 68) and men users 77% (*n* = 42). Non-users were respectively 38% for women (*n* = 41) and 24% for men (*n* = 13).

A non-parametric two-way ANOVA was performed to evaluate sex and age group differences in the total sample (Table 3). Regarding the sexes, significant differences were found in two items: “I prefer to do outdoor physical activity” and “I learn about local environmental issue from family/friends”. For the first item, men had higher scores than women, but for the second item, women had higher scores than men.

Regarding age groups, significant differences were found in the items “I prefer to do outdoor physical activity”, “Green space is important”, “Nature parks are boring”, “I learn about local environmental issue from family/friends”, and in the total score of the cognitive component and the total score of the affective components. Such differences were mostly found between the youngest age group (18–30 years) and the oldest groups (61–70 years and over 70). The participants of the age group 18–30 years generally showed lower values than the participants of the other age groups for many items. Significant interactions were observed between sexes and age groups in thirteen items. Women of all age groups generally presented higher scores than men; women aged 41–50 years showed lower scores than women aged 31–40 years.

When the distance of the dwelling from the park was considered, 69% of the participants indicated a distance of less than 300 m from their residence to the nearest park and only 31% indicated a greater distance. No significant differences were found in attitudes between the two groups.

Since one of the aims of the present study was to highlight any differences in the attitude towards green space between the participants of the two regions, we carried out a non-parametric ANOVA considering regions, sexes and age groups. In Table 4, the mean values and standard deviations of the considered items for regions, sexes and age groups are reported, while the ANOVA results are shown in Table 5.

With regard to the differences between regions, significant differences were found in 13 items (Table 5). Participants from Emilia-Romagna generally presented higher scores than those from Veneto. Numerous significant interactions were observed between regions and sexes: in general, men from Veneto had the lowest values while women from Emilia-Romagna had the highest. Considering regions and age groups, the highest values of the scores were observed in the oldest participants from Emilia-Romagna (61–70 years and over 70 years); the lowest scores were observed in the youngest participants from Veneto (18–30 years and 31–40 years). Regarding the interaction between the three factors (sexes, age groups and regions), the men from Veneto aged 31–40 years generally showed the lowest scores in nearly all the considered items. In particular, men from Veneto aged 31–40 showed significant differences in comparison with their peers in the items “Contact with nature is important to well-being” and “Tax dollars should be spent on nature parks”. In addition, they presented significant differences with the older women from Emilia-Romagna (61–70 years and over 70 years) in the items “Green space in cities is important”, “I expect to feel refreshed after visiting a nature park”, “I like the structure of the park I use” and the total score of the cognitive and affective components.

Multiple regression models were carried out to quantify the relationship between the dependent variable (the total score of the three components) and the explanatory variables (demographic characteristics). The results of the multiple regressions divided for the three different components are shown in Table 6, Table 7 and Table 8. The analysis was carried out first on the entire sample and then separately for Emilia-Romagna and Veneto. 

Table 6 shows the results for the cognitive component. The total model explained 27% of the variance. The results revealed that age group 18–30 years (regression coefficient, β = −0.20, *p* < 0.05), profession (employee β = −0.20, *p* < 0.005, managing director β = −0.20, *p* < 0.05, health care professional β = −0.21, *p* < 0.05) and do not use the park (β = −0.22, *p* < 0.05) were negative predictors of the total score of the cognitive component. For Emilia-Romagna, the model explained 44% of the variance and the results were similar to the total model. Age group 18–30 years (β = −0.43, *p* < 0.05), profession (employee β = −0.35, *p* < 0.05, managing director β = −0.25, *p* < 0.05) and do not use the park (β = −0.23, *p* < 0.05) demonstrated a negative relationship with the total score of the cognitive component. Instead, a bachelor’s degree (β = 2.67, *p* < 0.05) and the profession of engineer (β = 0.18, *p* < 0.05) were positive predictors of this component. For Veneto, the model explained 45% of the variance. The results revealed that profession (health care profession β = −0.33, *p* < 0.05) and do not use the park (β = −0.35, *p* < 0.05) were negative predictors of the cognitive component.

#### 3.2.2. Multiple Regression

Table 7 shows the result of the multiple regression for the behavioural component. The total model explained 23% of the variance. The results showed that a bachelor’s degree (β = 0.23, *p* < 0.05) was a positive predictor of the behavioural component, while professions (employee β = −0.08, *p* < 0.05, health care profession β = −0.14, *p* < 0.05) was a negative predictor. For Emilia-Romagna, the model explained 38% of the variance. The results revealed that a bachelor’s degree (β = 0.44, *p* < 0.05), the profession of lawyer (β = 0.24, *p* < 0.05) and living more than 300 m from the park (β = 0.21, *p* < 0.05) were positive predictors of the behavioural component. Moreover, the profession of employee (β = −0.35, *p* < 0.05) was a negative predictor of the behavioural component. For Veneto, the model explained 48% of the variance: being a health care professional (β = −0.44, *p* < 0.05) and not using the park (β = −0.36, *p* < 0.05) demonstrated a negative relationship with the behavioural component. 

Table 8 shows the results of the multiple regression for the affective component. The total model explained 32% of the variance. Belonging to the age group 18–31 years (β = −0.37, *p* < 0.05) and being an employee (β = −0.28, *p* < 0.05) showed a negative relation with the affective component. For Emilia-Romagna, the model explained 35% of the variance. Similar to the general model, the results showed that age group 18–31 years (β = −0.44, *p* < 0.05) and the profession of employee (β = −0.33, *p* < 0.05) were negative predictors of the affective component. However, having a bachelor’s degree (β = 0.20, *p* < 0.05), being a consultant (β = 0.18, *p* < 0.05) and living nearer than 300 m to a park (β = 0.05, *p* < 0.05) showed a positive relation with this component. In Veneto, the model explained 46% of the variance, and the profession of health care professionals (β = −0.39, *p* < 0.05) showed a negative relationship with the affective component. 

## 4. Discussion

The goals of this exploratory study were to develop a questionnaire to evaluate the attitude towards green space and to assess any differences between participants from two Italian regions: Emilia-Romagna and Veneto. To accomplish this goal, additional factors were considered. The questionnaire was developed in order to better understand what drives people to use green urban spaces and parks through the assessment of the attitude towards such spaces. The questionnaire consisted of fifteen items that investigated three components (cognitive, behavioural and affective) to gain a clear idea of what mostly influenced the attitude. The questionnaire was validated and seems to be an interesting tool to use in further investigation.

The two considered regions (Emilia-Romagna and Veneto) are both in the north of Italy and they are bordering each other, and similar in population characteristics and socio-economic status; however, they have different territorial policies. In fact, in Emilia-Romagna, a project was approved in 2020 to increase green areas in cities by 20%, while in Veneto there are no such projects [68]. In 2021, Emilia-Romagna allocated several millions for parks and biodiversity agencies and for projects protecting and enhancing the natural environment across the whole regional territory [57,58], eight areas of Emilia-Romagna were chosen by the Europarc Federation to participate in the “European charter for sustainable tourism” with the goal of developing environmental tourism [59]. In Bologna there are different projects and events to promote the green space. In fact, since 2005 the municipality has celebrated “Arbor Day”, where trees are planted in different parts of the city [60] and since 2010 they created the project “Parchi in movimento”, where citizens. had the possibility to experience various kinds of outdoor physical activity [61]. from 2011 onwards, senior citizens have had the possibility to participate in Bologna at the project “Badabene alla salute”, to be physically active towards walking groups in public parks. Similar projects were carried out in the other provinces of Emilia-Romagna and Veneto has had only projects linked to the local municipalities a (such as Padua or Verona), [62,63]. Higher scores in attitude towards green urban spaces could be a demonstration of the efficacy of the territorial policies in Emilia-Romagna.

We considered two other important demographic factors: sex and age. In the present study, women joined the project in a greater number than men. This is in accordance with the study by Smith et al. in which it was observed that women are more likely to have a greater willingness to participate in online surveys than men [69], and with the studies by Gascon et al. as well as van Praag et al. and Pattyn et al. [45,70,71] which found that women have a greater sensibility toward the neighbourhood environment. The results showed a different trend for women in Emilia-Romagna and Veneto. In fact, females from Emilia-Romagna had higher scores than females in Veneto. These differences could be interpreted in light of the different territorial policies implemented in the two regions.

Age was found to be one of the main factors influencing attitude. In fact, the subjects of the age group 18–31 years had the lowest score in many items and showed significant differences with the participants of older age groups; in addition, belonging to this age group was a negative predictor in the multiple regression analysis. This is in line with previous studies that showed that parks were generally underutilized by young people [72,73]. Young people tend to spend most of their leisure time on the Internet, rather than engaging in outdoor activities [47]. According to other studies, residents aged 20–30 are less likely to visit parks in their daily life because they prefer to pursue more active and exciting activities [74], or because they have less leisure time due to work and study commitments [75,76]. Moreover, according to Chen et al. [77] it is possible that young people could think that green urban spaces are occupied by older people and children and for this reason they may not be attracted to using the park. This is not in line with a nationwide study in Denmark which suggested that 91.5% of the adult population used green spaces at least once a week [61]. In Northern Europe, there are many green spaces containing more physically challenging facilities that could encourage young people to use them and to have a better attitude towards green spaces [78].

Several studies have highlighted the importance of the distance between home and the nearest park in influencing the attitude toward green spaces [40,79,80,81,82]. In this study, however, this aspect does not appear to have been such an important factor in influencing attitudes. In fact, the difference in the item scores between participants who lived nearer than 300 m to the park and those who lived farther than 300 m was not significant. Moreover, no significant correlation was found between the use of the park and the creation of an attitude; therefore, it seems that although people may understand the importance of green urban spaces, this may not be enough to drive them to use the park. Several studies have suggested that urbanization entails a lower level of attitude towards green spaces, resulting from a decrease in the level of interaction with nature and lower expectations on the quality of nature [2,83,84,85].

The cognitive component had a higher score than the other components, while the affective component demonstrated a weaker relationship with park attitude. This is in accordance with Wright et al. and Baur et al. who reported that the cognitive factor had a large statistically significant path coefficient to the creation of attitudes [86,87]. It is possible, therefore, to affirm that in the present study the cognitive component had a greater influence on the creation of an attitude towards green space. This leads one to suppose that attitude has a positive association with the logical, reasoned, conscious and purposeful evaluation of parks and their characteristics and utilization. The multiple regression analysis highlighted an important relationship between the different components and the professions. In particular, being an employee proved negatively related to the three components of the questionnaire, both in the total model and in the Emilia-Romagna model; this could be due to the high number of hours spent at work or less leisure time available compared to other professions. To our knowledge, no other studies have included the profession of participants, and this could be an important factor to consider in future research.

### Limits and Strengths

The questionnaire was administered throughout the whole of the regional territory, without taking into account the possible differences between the various provinces and places of living, for example, urban or more rural areas, and this could represent a limitation to the study. In addition, as an exploratory survey, the number of people involved was limited. Moreover, only two regions were included in the research, as a pilot study. Both regions are in the north of Italy and have similar socio-demographic characteristics.

Given the lack of adequate instruments to assess people’s attitude toward green space, the proposed questionnaire could represent an important new tool to better understand the factors that influence the person’s decision to use green urban spaces or parks. This could help local governments and organizations plan strategies to improve the population’s health. To the best of our knowledge, there are no similar surveys or research in Italy, and the present study could be an important starting point for future research. The evaluation and comparison among citizens from regions other than those considered here could provide a more complete framework of the territorial differences, linked to the geographic position or to territorial policies.

## 5. Conclusions

Understanding what drives people to use green urban spaces is a complex issue, especially due to its multifactorial nature. Results from this study suggest that territorial policies are fundamental to helping people understand the importance of green spaces. Even though national policies exist, the strategies adopted at a territorial level appear to be more effective. The results of the present study could be useful to local politicians in planning new measures to improve the use of green urban spaces. The age class seems to be an important predictor, as the attitude score increases with increasing age. For this reason, local politicians could create events or projects to engage specifically with the subjects belonging to the younger age groups (18–30). These might be cultural or sport events aimed at changing the perception that young people have about parks. Another possibility could be to increase the quality of the parks, by adding outdoor fitness equipment, wellness paths or other facilities (such as toilets or benches). Therefore, awareness must be promoted at all ages. In addition, the type of work seems to be an important predictor of the use of parks, and this is an aspect to consider in future studies. Local politicians could plan and design peri-urban business sites and design interventions to promote employees’ well-being. They should create restorative workplace environments, in order to meet the needs of workers. A better understanding of the relationship between the creation of attitudes and the kind of profession or the number of hours spent at work is needed. Finally, understanding people’s attitudes will help to improve the quality of life in cities by creating affordable parks and green spaces for the entire population.

## Figures and Tables

**Table 1:** Reliability analysis.

Attitude Components	Items	Loading Value	Cronbach’s α
Cognitive component	I prefer to do outdoor physical activity	0.965	
	Green space in cities is important	0.898	
	Nature parks improve quality of life	0.897	0.929
	Contact with nature is important for well-being	0.893	
	It is important to have convenient nature parks in cities	0.910	
Behavioural component	Nature parks are boring	0.793	
	Humans have the right to modify nature to suit our needs	0.623	
	The time spent in an urban nature park relaxes you	0.592	
	Tax dollars should be spent on nature parks	0.637	0.704
	Nature parks in the cities provide valuable contacts with nature	0.583	
Affective components	I expect to feel refreshed after visiting a nature park	0.750	
	I enjoy talking with neighbours at local nature park	0.748	
	I learn about local environmental issue from friends/family	0.787	0.761
	I like the structure of the park you use	0.657	
	I can count on family and friends for help	0.710	

**Table 2:** Demographic characteristics of participants (*n* = 310) and Chi-square test between the frequencies of the two regions.

	Emilia-Romagna		Veneto			
Characteristics	Male	Female	Male	Female	χ^2^	*p*
Age	*n* (%)	*n* (%)	*n* (%)	*n* (%)	32.59	<0.001
18–31	13 (22.4%)	18 (16.5%)	13 (22.0%)	23 (27.4%)		
31–40	9 (15.5%)	17 (15.6%)	14 (23.7%)	11 (13.1%)		
41–50	6 (10.3%)	10 (9.2%)	12 (20.3%)	21 (25.0%)		
51–60	12 (20.7%)	30 (27.5%)	14 (23.7%)	24 (28.6%)		
61–70	12 (20.7%)	25 (22.9%)	5 (8.5%)	5 (6.0%)		
Over 70	6 (10.3%)	9 (8.3%)	1 (1.7%)			
Education Level					32.32	<0.001
Below high school	6 (10.3%)	15 (13.8%)	3 (5.1%)	3 (3.6%)		
High school	9 (15.5%)	35 (32.1%)	27 (45.8%)	41 (48.8%)		
Bachelor’s degree	6 (10.3%)	8 (7.3%)	9 (15.3%)	14 (16.7%)		
Master’s degree	24 (41.4%)	41 (37.6%)	19 (32.2%)	23 (27.4%)		
Doctorate	13 (22.4%)	10 (9.2%)	1 (1.7%)	3 (3.6%)		
Marital status					10.16	<0.05
Single	18 (31.0%)	34 (31.2%)	11 (18.6%)	24 (28.6%)		
Engaged	3 (5.2%)	7 (6.4%)	6 (10.2%)	12 (14.3%)		
Cohabiting	6 (10.3%)	9 (8.3%)	6 (10.2%)	12 (14.3%)		
Married	29 (50.0%)	56 (51.4%)	36 (61.0%)	36 (42.9%)		
Widower	1 (1.7%)	4 (3.7%)				
Distance from park					8.26	<0.05
Less than 300 m	44 (17.2%)	81 (29.4%)	40 (32.2%)	48 (42.9%)		
More than 300 m	10 (82.8%)	32 (70.6%)	19 (67.8%)	36 (57.1%)		
Use of the park					0.07	0.70
Users	41 (74.1%)	71 (63.3%)	39 (66.1%)	55 (65.5%)		
Non-users	13 (25.9%)	42 (36.7%)	20 (33.9%)	29 (34.5%)		

Note. Some demographic characteristics were no present in all the sample (female over 70 years in Veneto, widower in Veneto).

**Table 3:** Descriptive statistics and ANOVA for sexes and age groups.

	18–30 Years		31–40 Years		41–50 Years		51–60 Years		61–70 Years		Over 70 Years							
	Male	Female	Male	Female	Male	Female	Male	Female	Male	Female	Male	Female	Sexes		Age		Sexes*Age	
	Mean (SD)	Mean (SD)	Mean (SD)	Mean (SD)	Mean (SD)	Mean (SD)	Mean (SD)	Mean (SD)	Mean (SD)	Mean (SD)	Mean (SD)	Mean (SD)	F	*p*	F	*p*	F	*p*
I prefer to do outdoor physical activity	3.84 (1.21)	3.56 (1.36)	3.70 (1.33)	3.82 (1.42)	4.56 (0.78)	3.39 (1.45)	4.31 (0.97)	3.89 (1.25)	4.35 (1.00)	4.11 (1.40)	4.86 (0.38)	4.50 (0.53)	6.30	<0.05	2.45	<0.05	2.38	<0.05
Green space in cities is important	4.44 (0.82)	4.29 (0.96)	3.87 (1.25)	4.64 (0.78)	4.72 (0.57)	3.68 (1.30)	4.58 (0.81)	4.38 (0.84)	4.65 (0.61)	4.44 (0.89)	5.00 (0.00)	4.75 (0.46)	1.81	0.18	2.39	<0.05	3.54	<0.05
Nature parks improve quality of life	4.16 (0.99)	4.10 (0.97)	4.09 (1.28)	4.82 (0.55)	4.89 (0.32)	3.74 (1.15)	4.42 (0.86)	4.32 (0.92)	4.76 (0.56)	4.48 (0.89)	5.00 (0.00)	4.88 (0.35)	1.86	0.17	3.32	0.06	4.20	<0.05
Contact with nature is important for well-being	4.40 (0.76)	4.37 (0.89)	4.00 (1.24)	4.79 (0.57)	4.78 (0.55)	4.00 (1.06)	4.50 (0.86)	4.39 (0.93)	4.76 (0.56)	4.44 (0.89)	4.86 (0.38)	4.75 (0.46)	0.43	0.51	1.20	0.31	2.47	<0.05
It is important to have convenient nature parks in cities	4.32 (0.85)	4.29 (0.87)	4.13 (1.25)	4.80 (0.57)	4.83 (0.71)	4.00 (1.07)	4.35 (0.89)	4.41 (0.91)	4.76 (0.56)	4.48 (0.89)	4.71 (0.49)	4.88 (0.35)	0.25	0.61	1.33	0.25	2.33	<0.05
Nature parks are boring	1.96 (0.98)	1.71 (1.25)	1.26 (0.45)	1.14 (0.45)	1.28 (0.46)	1.48 (0.96)	1.38 (0.57)	1.55 (1.06)	1.35 (0.70)	1.11 (0.42)	1.86 (1.57)	1.75 (1.49)	0.11	0.74	4.12	<0.05	2.18	<0.05
Humans have the right to modify nature to suit our needs	2.12 (0.88)	1.95 (1.05)	1.70 (0.76)	1.68 (0.82)	1.83 (1.10)	1.71 (0.78)	1.85 (1.05)	1.66 (1.05)	2.00 (1.32)	1.70 (0.91)	2.14 (1.46)	2.50 (1.69)	1.43	0.23	1.66	0.15	0.99	0.45
The time spent in an urban nature park relaxes you	4.00 (1.08)	3.88 (1.05)	3.65 (1.23)	4.50 (0.92)	4.33 (0.77)	3.77 (1.09)	3.62 (1.30)	4.20 (1.02)	4.24 (0.75)	4.19 (1.11)	4.29 (0.76)	4.75 (0.46)	0.55	0.46	1.20	0.31	2.22	<0.05
Tax dollars should be spent on nature parks	4.32 (3.76)	4.39 (0.83)	4.09 (1.24)	4.79 (0.57)	4.94 (0.24)	4.00 (1.13)	4.38 (0.85)	4.39 (0.85)	4.76 (0.56)	4.33 (1.11)	4.86 (0.38)	4.88 (0.35)	1.79	0.18	1.01	0.41	2.64	<0.05
Nature parks in the cities provide valuable contacts with nature	3.76 (1.09)	3.80 (1.12)	3.61 (1.20)	4.25 (1.00)	4.22 (1.11)	3.74 (1.09)	4.04 (0.96)	4.18 (1.05)	4.12 (0.93)	4.33 (0.92)	3.86 (1.07)	4.63 (0.52)	2.25	0.13	1.70	0.13	1.71	0.07
I expect to feel refreshed after visiting a nature park	3.76 (1.09)	3.73 (1.03)	3.35 (1.15)	4.18 (1.09)	4.22 (1.11)	3.58 (1.09)	3.58 (1.24)	4.20 (1.05)	4.18 (0.81)	4.11 (1.05)	4.14 (1.07)	4.63 (0.52)	2.70	0.10	1.19	0.09	2.24	<0.05
I enjoy talking with neighbours at local nature park	2.28 (1.14)	2.29 (1.23)	3.61 (1.22)	4.25 (1.00)	4.11 (1.08)	3.76 (1.09)	4.04 (0.96)	4.09 (1.03)	4.14 (0.81)	4.35 (0.94)	3.86 (1.05)	4.75 (0.46)	0.84	0.36	2.24	0.06	2.36	<0.05
I learn about local environmental issue from friends/family	2.36 (1.25)	3.80 (1.12)	2.35 (1.19)	2.57 (1.23)	3.11 (1.13)	2.74 (1.32)	2.38 (1.24)	2.70 (1.43)	2.59 (0.80)	2.85 (1.46)	2.86 (1.68)	3.50 (1.07)	7.83	<0.05	2.25	<0.05	1.37	0.18
I like the structure of the park I use	3.16 (0.90)	2.61 (1.38)	2.74 (1.05)	3.25 (1.08)	3.11 (1.23)	2.90 (1.14)	3.31 (1.19)	3.50 (1.21)	3.76 (1.25)	3.56 (1.37)	3.86 (1.21)	3.88 (1.55)	0.02	0.88	4.83	<0.05	2.41	<0.05
I can count on family and friends for help	2.28 (1.14)	1.73 (1.05)	2.30 (1.49)	2.14 (1.51)	2.44 (1.10)	1.58 (0.81)	2.54 (1.17)	1.95 (1.21)	2.35 (1.00)	2.56 (1.45)	2.57 (1.72)	3.25 (1.58)	2.69	0.37	1.38	0.10	1.21	0.28
Total score of cognitive	21.16 (4.10)	20.61 (4.29)	19.78 (5.83)	22.86 (3.23)	23.78 (1.96)	18.81 (5.44)	22.15 (3.78)	21.39 (4.50)	23.29 (2.69)	21.96 (4.85)	24.43 (1.13)	23.75 (1.67)	2.34	0.13	2.30	<0.05	3.28	<0.05
Total score of behavioural	16.16 (2.91)	15.73 (3.35)	14.30 (3.71)	16.36 (2.53)	16.61 (2.03)	14.71 (3.40)	15.27 (3.38)	15.98 (3.25)	16.47 (2.55)	15.67 (2.94)	17.00 (3.87)	18.50 (2.33)	0.07	0.80	1.57	0.16	1.85	<0.05
Total score of affective	15.32 (3.65)	14.17 (3.88)	14.35 (4.84)	16.39 (3.45)	17.00 (3.99)	14.55 (4.13)	15.85 (4.51)	16.41 (4.61)	17.00 (2.35)	17.41 (5.15)	17.29 (5.28)	20.00 (2.73)	0.20	0.66	3.52	<0.05	2.72	<0.05

Note. F = test F, *p* = *p*-value, * = interaction between the two variables.

**Table 4:** Descriptive statistics in Emilia-Romagna and Veneto for age groups and sexes.

	Emilia-Romagna												Veneto									
	18–30 Years		31–40 Years		41–50 Years		51–60 Years		61–70 Years		Over 70 Years		18–30 Years		31–40 Years		41–50 Years		51–60 Years		61–70 Years	
	M	F	M	F	M	F	M	F	M	F	M	F	M	F	M	F	M	F	M	F	M	F
I prefer to do outdoor physical activity	3.25 (1.66)	3.21 (1.55)	4.10 (1.60)	3.56 (1.54)	3.86 (1.86)	2.82 (1.94)	3.92 (1.44)	3.76 (1.41)	4.00 (1.58)	4.50 (1.21)	4.83 (0.41)	4.44 (1.49)	3.79 (1.53)	3.54 (1.53)	2.93 (1.53)	3.69 (1.93)	4.29 (1.51)	3.43 (1.47)	4.07 (0.53)	3.76 (1.45)	3.67 (1.97)	2.00 (2.00)
Green space in cities is important	3.67 (1.50)	4.16 (1.34)	4.10 (1.66)	4.28 (1.41)	4.00 (1.83)	3.27 (1.90)	4.15 (1.52)	4.36 (1.08)	4.23 (1.42)	4.65 (1.16)	5.00 (0.00)	4.67 (1.55)	4.43 (1.34)	4.04 (1.30)	3.20 (1.52)	4.46 (1.44)	4.43 (1.49)	3.61 (1.37)	4.33 (1.40)	4.04 (1.24)	4.00 (1.00)	3.17 (1.83)
Nature parks improve quality of life	3.42 (1.56)	4.05 (1.22)	4.20 (1.62)	4.50 (1.29)	4.00 (1.83)	3.64 (1.57)	3.85 (1.46)	4.33 (1.14)	4.38 (1.45)	4.69 (1.16)	5.00 (0.00)	4.78 (1.57)	4.14 (1.35)	3.79 (1.35)	3.47 (1.68)	4.54 (1.45)	4.64 (1.46)	3.52 (1.41)	4.13 (0.41)	3.96 (1.27)	4.00 (1.00)	3.17 (1.83)
Contact with nature is important for well-being	3.75 (1.48)	4.26 (1.28)	4.20 (1.62)	4.44 (1.29)	3.86 (1.86)	4.18 (1.60)	4.00 (1.53)	4.33 (1.22)	4.38 (1.45)	4.69 (1.16)	4.83 (0.41)	4.67 (1.55)	4.29 (1.33)	4.08 (1.28)	3.33 (1.59)	4.54 (1.45)	4.57 (0.36)	3.61 (1.30)	4.33 (0.40)	4.12 (1.24)	4.00 (1.00)	3.15 (1.83)
It is important to have convenient nature parks in cities	3.75 (1.48)	4.21 (1.27)	4.40 (1.58)	4.44 (1.29)	3.86 (2.04)	4.18 (1.60)	3.92 (1.50)	4.33 (1.19)	1.08 (0.49)	4.65 (1.16)	4.83 (0.41)	4.78 (1.57)	4.14 (1.41)	4.00 (1.25)	3.40 (1.64)	4.54 (1.45)	4.64 (0.26)	3.60 (1.31)	1.27 (0.59)	4.16 (1.25)	3.67 (1.95)	3.17 (1.80)
Nature parks are boring	1.75 (1.22)	1.68 (1.42)	1.20 (0.63)	1.00 (0.34)	1.00 (0.58)	1.45 (1.04)	1.31 (0.75)	1.64 (1.14)	1.69 (1.25)	1.12 (0.47)	1.67 (1.63)	1.67 (1.43)	1.93 (1.00)	1.58 (1.18)	1.13 (0.52)	1.15 (0.72)	1.21 (0.59)	1.35 (1.00)	1.47 (0.74)	1.32 (0.99)	1.50 (1.22)	0.83 (0.41)
Humans have the right to modify nature to suit our needs	2.08 (1.08)	2.05 (1.35)	1.50 (0.97)	1.50 (0.86)	1.86 (1.68)	1.55 (0.69)	2.00 (1.41)	1.55 (1.03)	4.00 (1.35)	1.77 (0.95)	2.33 (1.51)	2.78 (1.90)	1.86 (1.03)	1.71 (0.86)	1.60 (0.83)	1.62 (0.98)	1.50 (0.83)	1.61 (0.95)	3.40 (1.80)	1.68 (1.14)	2.00 (1.79)	1.00 (0.63)
The time spent in an urban nature park relaxes you	3.75 (1.42)	3.74 (1.37)	3.80 (1.62)	4.11 (1.45)	3.43 (1.72)	3.91 (1.51)	3.31 (1.32)	4.06 (1.27)	4.38 (1.45)	4.38 (1.34)	4.50 (0.55)	4.67 (1.55)	3.57 (1.60)	3.67 (1.31)	3.07 (1.49)	4.23 (1.50)	4.21 (1.36)	3.43 (1.33)	4.27 (0.39)	4.04 (1.27)	3.33 (1.86)	3.17 (1.83)
Tax dollars should be spent on nature parks	3.75 (1.42)	4.16 (1.26)	4.40 (1.58)	4.50 (1.29)	4.14 (1.86)	4.18 (1.60)	3.85 (1.46)	4.39 (1.09)	4.00 (1.35)	4.54 (1.33)	5.00 (0.00)	4.78 (1.57)	4.21 (1.37)	4.21 (1.25)	3.33 (1.59)	4.46 (1.44)	4.64 (0.46)	3.61 (1.37)	3.80 (1.42)	4.04 (1.24)	4.00 (1.00)	3.17 (1.83)
Nature parks in the cities provide valuable contacts with nature	3.67 (1.56)	3.84 (1.46)	3.70 (1.64)	3.83 (1.38)	3.57 (2.15)	4.00 (1.55)	3.69 (1.44)	4.03 (1.36)	4.38 (1.45)	4.54 (1.18)	4.17 (0.75)	4.56 (1.52)	3.43 (1.45)	3.46 (1.28)	3.07 (1.44)	4.23 (1.59)	4.00 (1.00)	3.30 (1.28)	3.80 (1.50)	4.04 (1.21)	3.00 (1.90)	3.17 (1.83)
I expect to feel refreshed after visiting a nature park	3.42 (1.51)	4.05 (1.39)	3.50 (1.51)	4.06 (1.30)	3.43 (2.07)	3.73 (1.62)	3.69 (1.44)	4.03 (1.36)	4.00 (1.35)	4.54 (1.11)	3.17 (1.47)	4.44 (1.56)	3.43 (1.45)	3.67 (1.31)	2.80 (1.37)	4.23 (1.59)	4.14 (0.31)	3.30 (1.28)	3.33 (1.72)	4.00 (1.00)	3.00 (1.85)	3.00 (1.67)
I enjoy talking with neighbours at local nature park	3.92 (1.44)	3.95 (1.31)	3.40 (1.43)	3.67 (1.24)	2.00 (1.41)	3.82 (1.47)	3.31 (1.32)	4.06 (1.27)	3.54 (1.51)	4.46 (1.17)	3.33 (1.21)	4.44 (1.56)	3.36 (1.50)	3.46 (1.28)	2.00 (1.31)	4.00 (1.51)	4.00 (1.00)	3.30 (1.30)	2.40 (1.30)	2.56 (1.45)	3.17 (1.83)	3.17 (1.83)
I learn about local environmental issue from friends/family	3.75 (1.42)	3.11 (1.37)	1.90 (0.99)	2.22 (1.22)	2.14 (1.35)	1.91 (1.51)	3.31 (1.32)	3.91 (1.23)	3.38 (1.61)	3.54 (1.42)	1.83 (1.17)	3.44 (1.60)	1.79 (1.12)	2.00 (1.06)	3.07 (1.44)	2.54 (1.61)	2.86 (1.25)	2.57 (1.41)	2.47 (1.36)	1.56 (1.46)	2.17 (1.33)	1.50 (1.38)
I like the structure of the park I use	2.58 (1.44)	2.58 (1.84)	1.90 (1.20)	1.61 (1.42)	3.14 (1.95)	2.18 (1.54)	3.23 (1.36)	3.58 (1.41)	2.38 (1.45)	3.42 (1.51)	2.17 (1.17)	3.33 (1.83)	2.79 (1.31)	1.79 (0.88)	2.20 (1.08)	2.38 (1.70)	2.29 (1.25)	1.70 (0.90)	2.93 (1.53)	3.12 (1.24)	2.50 (1.64)	1.17 (0.75)
I can count on family and friends for help	3.33 (1.72)	3.21 (1.40)	2.90 (1.20)	3.17 (1.42)	3.71 (1.80)	3.55 (1.57)	2.00 (1.41)	2.12 (1.41)	1.69 (1.25)	3.12 (1.57)	3.67 (1.51)	2.11 (1.29)	1.79 (1.12)	2.21 (1.22)	1.87 (1.25)	3.15 (1.47)	3.00 (1.52)	2.52 (1.18)	3.80 (1.42)	2.57 (1.60)	2.67 (1.97)	1.50 (1.22)
Total score of cognitive	17.83 (7.49)	19.89 (6.24)	21.00 (7.76)	21.22 (1.54)	19.57 (9.09)	18.09 (7.78)	19.85 (7.12)	21.12 (5.73)	21.38 (7.02)	23.19 (5.76)	24.50 (1.22)	23.33 (7.62)	20.79 (6.44)	19.46 (6.06)	16.33 (7.54)	21.77 (7.18)	22.57 (6.88)	17.78 (6.70)	21.20 (6.81)	20.04 (6.21)	19.67 (9.83)	14.67 (9.14)
Total score of behavioural	14.67 (5.14)	15.47 (5.44)	14.60 (5.38)	14.94 (4.53)	14.00 (6.88)	15.09 (5.52)	14.15 (5.40)	15.67 (4.20)	15.15 (5.06)	16.35 (3.98)	17.67 (3.78)	18.44 (6.19)	15.00 (5.45)	14.63 (4.07)	12.20 (5.24)	15.69 (5.38)	15.57 (4.64)	13.30 (4.48)	14.20 (5.13)	15.12 (4.59)	13.83 (7.41)	11.33 (6.41)
Total score of affective	19.25 (7.61)	20.74 (7.55)	17.00 (6.80)	17.28 (6.32)	18.00 (9.80)	19.18 (7.92)	17.77 (6.85)	20.33 (7.03)	17.62 (7.68)	21.23 (8.62)	18.00 (5.25)	18.78 (9.86)	13.14 (5.02)	13.13 (4.05)	11.93 (5.35)	16.31 (6.10)	16.29 (5.54)	13.39 (5.14)	14.93 (6.51)	15.28 (4.86)	13.50 (7.01)	10.33 (6.62)

Note. M = male, F = female.

**Table 5:** Results of interaction of regions, sexes and age groups of non-parametric ANOVA.

	Regions		Age*Regions		Sexes*Regions		Age*Sexes*Regions	
	F	*p*	F	*p*	F	*p*	F	*p*
I prefer to do outdoor physical activity	1.65	0.20	2.06	<0.05	2.84	<0.05	2.26	<0.05
Green space in cities is important	3.85	0.05	1.57	0.10	2.36	0.07	2.67	<0.05
Nature parks improve quality of life	6.43	<0.05	1.98	<0.05	4.85	<0.05	3.12	<0.05
Contact with nature is important for well-being	6.70	<0.05	1.33	0.20	3.42	<0.05	2.51	<0.05
It is important to have convenient nature parks in cities	8.50	<0.05	1.47	0.14	3.53	<0.05	2.50	<0.05
Nature parks are boring	0.01	0.91	2.25	<0.05	0.20	0.89	1.39	0.12
Humans have the right to modify nature to suit our needs	1.55	0.21	1.15	0.32	1.29	0.28	0.94	0.55
The time spent in an urban nature park relaxes you	6.36	<0.05	1.16	0.31	2.71	<0.05	1.77	<0.05
Tax dollars should be spent on nature parks	7.29	<0.05	1.73	0.06	3.24	<0.05	2.78	<0.05
Nature parks in the cities provide valuable contacts with nature	9.88	<0.05	2.16	<0.05	3.73	<0.05	1.83	<0.05
I expect to feel refreshed after visiting a nature park	6.36	<0.05	1.16	0.31	2.72	<0.05	1.77	<0.05
I enjoy talking with neighbours at local nature park	4.29	<0.05	1.74	0.06	2.31	0.07	2.10	<0.05
I learn about local environmental issue from friends/family	6.10	<0.05	2.01	<0.05	2.41	0.06	1.31	<0.05
I like the structure of the park I use	27.04	<0.05	4.57	<0.05	9.27	<0.05	3.22	<0.05
I can count on family and friends for help	3.65	0.06	1.45	0.15	4.23	<0.05	2.12	<0.05
Total score of cognitive	5.98	<0.05	1.75	0.06	3.60	<0.05	2.80	<0.05
Total score of behavioural	9.25	<0.05	1.56	0.11	3.10	<0.05	1.83	<0.05
Total score of affective	15.82	<0.05	3.28	<0.05	5.30	<0.05	2.73	<0.05

Note. * = interaction between the variables, F = test F, *p* = *p*-value.

**Table 6:** Multiple regression model for the cognitive component.

	Total Model			Emilia-Romagna			Veneto		
	β	T	*p*	β	T	*p*	β	T	*p*
Age									
18–30	−0.24	−2.11	<0.05	−0.43	−3.20	<0.05	0.00	0.00	0.99
31–40	−0.08	−1.01	0.31	0.25	0.20	0.84	−0.28	−1.52	0.13
41–50	0.00	−0.81	0.42	−0.04	−0.49	0.63	−0.03	−0.12	0.91
51–60	−0.02	−0.04	0.97	−0.14	−1.43	0.16	−0.07	−0.34	0.73
61–70	0.10	1.11	0.27	0.14	1.23	0.22	0.02	−0.10	0.91
Over 70	0.15	1.90	0.06	0.14	1.17	0.09			
Sexes									
Male	−0.04	−0.56	0.58	−1.29	−1.49	0.14	−0.09	−0.61	0.54
Marital status									
Single	−0.09	−1.04	0.30	−0.10	−1.14	0.26	−0.11	−0.80	0.43
Engaged	0.14	1.50	0.14	0.05	0.43	0.66	−0.03	−0.17	0.86
Cohabiting	0.01	0.09	0.93	−0.05	−0.52	0.61	0.15	1.06	0.30
Married	0.07	0.74	0.46	0.11	1.12	0.26	0.22	1.25	0.22
Education level									
Below high school	−0.04	−0.46	0.64	−0.14	−0.89	0.38	0.10	0.44	0.66
High school	−0.03	−0.30	0.77	0.06	0.56	0.57	−0.32	−1.49	0.14
Bachelor’s degree	0.15	1.53	0.13	0.31	2.67	<0.05	0.03	0.13	0.90
Master’s degree	−0.05	−0.60	0.55	−0.06	−0.77	0.44	−0.25	−1.30	0.20
Doctorate	0.00	−0.04	0.97	−0.05	−0.34	0.73			
Profession									
Freelance	0.02	0.34	0.74	−0.04	−0.38	0.70	0.15	1.16	0.25
Sport employee	0.01	0.12	0.90	−0.02	−0.17	0.86	0.09	0.67	0.50
Employee	−0.20	−2.46	<0.05	−0.35	−3.50	<0.05	−0.03	−0.25	0.80
Engineer	0.08	1.08	0.28	0.18	2.07	<0.05	0.02	0.09	0.92
Managing director	−0.20	−2.23	<0.05	−0.25	−2.20	<0.05	−0.15	−0.89	0.38
Teacher	0.02	0.20	0.84	0.02	0.26	0.80	0.05	0.31	0.76
Doctor	−0.01	−0.18	0.86	0.02	0.30	0.76	−0.25	1.38	0.17
Retired	0.11	−0.93	0.35	−0.20	−1.37	0.17	−0.14	−0.47	0.63
Consultant	−0.04	−0.53	0.60	0.10	0.85	0.40	−0.02	0.53	0.60
Business owner	−0.09	−1.18	0.24	0.11	1.29	0.20	−0.20	−1.41	0.16
Unemployed	−0.04	−0.51	0.61	0.06	0.63	0.53	−0.22	−1.43	0.16
Worker	−0.01	−0.11	0.91	−0.13	−1.37	0.17	0.12	0.70	0.48
Lawyer	0.09	1.22	0.23	0.07	0.73	0.46	0.07	0.38	0.70
Health care professional	−0.21	−2.63	<0.05				−0.33	−2.02	<0.05
Park distance									
Less than 300 m	−0.03	−0.45	0.65	0.10	1.19	0.24	−0.69	−0.47	0.63
Park use									
No	−0.22	−2.98	<0.05	−0.23	−2.43	<0.05	−0.36	−2.36	<0.05
R^2^	0.27			0.44			0.45		
Adjusted R^2^	0.05			0.22			0.02		
*p*	0.16			<0.05			0.43		

Note. β = regression coefficient, T = t-student, *p* = *p*-value, R^2^ = proportion of variance explained.

**Table 7:** Multiple regression for the behavioural component.

	Total Model			Emilia-Romagna			Veneto		
	β	T	*p*	β	T	*p*	β	T	*p*
Age									
18–30	−0.13	−1.79	0.07	−0.25	−1.78	0.08	−0.18	−0.83	0.41
31–40	−0.15	−1.47	0.14	−0.11	−0.99	0.32	−0.27	−1.50	0.14
41–50	−0.10	0.15	0.88	0.13	1.25	0.22	−0.13	−0.66	0.51
51–60	−0.03	0.17	0.87	−0.12	−1.02	0.31	−0.05	−0.25	0.80
61–70	−0.03	−0.07	0.94	−0.04	−0.37	0.71	0.05	0.33	0.73
Over 70	0.11	1.13	0.26	0.18	1.80	0.08			
Sexes									
Male	−0.02	−0.73	0.47	−0.02	−0.32	0.74	−0.13	−0.90	0.38
Marital status									
Single	−0.10	−1.50	0.14	0.10	−1.90	0.06	−0.12	−0.86	0.40
Engaged	0.02	0.66	0.51	0.13	1.14	0.26	−0.11	−0.65	0.52
Cohabiting	−0.10	−0.38	0.70	−0.10	−0.90	0.37	0.01	0.07	0.94
Married	0.09	0.67	0.50	0.10	0.94	0.34	0.13	0.76	0.45
Education level									
Below high school	−0.03	−1.18	0.24	−0.16	−1.34	0.18	0.14	0.65	0.52
High school	0.07	0.53	0.59	0.18	1.68	0.10	−0.12	−0.59	0.56
Bachelor’s degree	0.23	2.87	<0.05	0.44	3.37	<0.05	0.25	1.17	0.24
Master’s degree	0.01	−0.22	0.82	−0.19	−1.62	0.11	−0.08	−0.44	0.66
Doctorate	−0.05	−0.56	0.58	−0.14	−0.90	0.37			
Profession									
Freelance	−0.04	−0.48	0.63	−0.18	−1.77	0.08	0.12	0.91	0.37
Sport employee	0.11	1.07	0.29	0.60	0.58	0.57	0.17	1.22	0.23
Employee	−0.08	−2.05	<0.05	−0.23	−2.37	<0.05	−0.09	−0.71	0.48
Engineer	0.02	1.05	0.29	0.08	0.88	0.38	0.12	0.71	0.48
Managing director	−0.15	−1.90	0.06	−0.13	−1.12	0.27	−0.23	−1.35	0.18
Teacher	0.07	0.71	0.48	0.05	0.50	0.62	0.09	0.67	0.51
Doctor	−0.01	−0.23	0.82	0.03	0.43	0.67	−0.03	−0.16	0.87
Retired	0.03	−0.27	0.82	0.00	−0.01	0.99	−0.37	−1.24	0.22
Consultant	−0.10	−1.00	0.32	0.06	0.50	0.62	−0.12	1.05	0.30
Business owner	−0.02	−0.95	0.34	0.08	0.92	0.36	−0.16	−1.18	0.24
Unemployed	−0.03	−0.27	0.78	0.06	0.60	0.55	−0.22	−1.48	0.15
Worker	−0.06	−0.77	0.44	−0.13	−1.39	0.17	−0.05	−0.30	0.77
Lawyer	0.14	1.76	0.08	0.24	2.32	<0.05	0.08	0.48	0.63
Health care professional	−0.14	−2.38	<0.05				−0.44	−2.70	<0.05
Park distance									
Less than 300 m	0.03	0.46	0.65	0.21	2.31	<0.05	−0.16	−1.09	0.28
Park use									
No	−0.07	−1.41	0.16	0.04	0.21	0.83	−0.36	−2.41	<0.05
R^2^	0.23			0.38			0.48		
Adjusted R^2^	0.03			0.13			0.06		
*p*	0.25			<0.05			0.33		

Note. β = regression coefficient, T = t-student, *p* = *p*-value, R^2^ = proportion of variance explained.

**Table 8:** Multiple regression for the affective component.

	Total Model			Emilia-Romagna			Veneto		
	β	T	*p*	β	T	*p*	β	T	*p*
Age									
18–30	−0.37	−3.41	<0.05	−0.44	−3.60	<0.05	−0.17	−0.77	0.44
31–40	−0.05	−0.69	0.49	−0.02	−0.40	0.69	−0.05	−0.25	0.80
41–50	0.08	−0.54	0.59	0.08	1.28	0.20	0.08	0.40	0.69
51–60	0.00	0.88	0.38	0.02	−1.97	0.05	0.18	0.92	0.36
61–70	0.08	0.93	0.35	0.08	1.27	0.21	0.11	0.71	0.48
Over 70	0.10	1.30	0.19	0.09	1.76	0.08			
Sexes									
Male	−0.02	−0.28	0.78	0.00	−0.72	0.47	−0.06	−0.36	0.71
Marital status									
Single	−0.09	−1.08	0.28	0.10	−1.32	0.19	−0.12	−0.85	0.40
Engaged	0.13	1.45	0.15	0.17	0.92	0.36	−0.02	−0.10	0.92
Cohabiting	−0.06	−0.69	0.49	0.03	−0.92	0.36	0.05	0.35	0.72
Married	−0.03	−0.26	0.79	0.00	−0.37	0.71	0.23	1.29	0.20
Education level									
Below high school	0.01	0.16	0.88	−0.20	−0.73	0.47	0.14	0.60	0.55
High school	0.05	0.59	0.55	−0.00	1.67	0.10	−0.07	−0.34	0.73
Bachelor’s degree	0.18	1.89	0.06	0.20	3.10	<0.05	0.08	0.37	0.71
Master’s degree	−0.05	−0.57	0.57	0.01	−1.42	0.16	−0.08	−0.44	0.66
Doctorate	−0.14	−1.23	0.22	−0.20	−1.66	0.10			
Profession									
Freelance	0.00	−0.01	0.99	−0.07	−1.09	0.28	0.12	0.93	0.36
Sport employee	0.06	0.80	0.43	0.05	0.61	0.54	0.14	0.99	0.32
Employee	−0.23	−2.99	<0.05	−0.33	−4.37	<0.05	−0.02	−0,13	0.90
Engineer	0.02	0.31	0.76	0.02	0.90	0.37	−0.06	−0.35	0.72
Managing director	−0.11	−1.31	0.19	−0.11	−0.34	0.73	−0.30	1.69	0.09
Teacher	−0.11	0.98	0.33	−0.00	−0.07	0.95	0.15	0.99	0.32
Doctor	0.07	−0.01	0.99	0.04	0.31	0.76	0.70	0.39	0.70
Retired	−0.14	−1.25	0.21	−0.26	−1.27	0.21	−0.26	−0.89	0.38
Consultant	−0.05	−0.70	0.49	0.18	2.08	<0.05	−0.15	0.86	0.39
Business owner	−0.12	−1.74	0.08	0.00	0.76	0.45	−0.24	−1.76	0.08
Unemployed	−0.10	−1.42	0.16	−0.06	−1.07	0.29	−0.22	−1.49	0.14
Worker	0.00	−0.01	0.99	−0.01	−0.31	0.76	0.04	0.24	0.80
Lawyer	0.06	0.82	0.41	0.05	0.15	0.88	0.09	0.54	0.58
Health care professional	−0.15	−1.88	0.06				−0.39	−2.36	<0.05
Park distance									
Less than 300 m	−0.04	−0.58	0.56	0.05	2.18	<0.05	−0.21	−1.42	0.16
Park use									
No	−0.12	−1.68	0.09	−0.13	0.78	0.44	−0.19	−1.26	0.21
R^2^	0.32			0.35			0.46		
Adjusted R^2^	0.11			0.10			0.03		
*p*	<0.05			0.09			0.40		

Note. β = regression coefficient, T = t-student, *p* = *p*-value, R^2^ = proportion of variance explained.
